# Terrestrial gastropods from caves in Serra dos Carajás, eastern Brazilian Amazon, with the description of two new species (Eupulmonata, Stylommatophora)

**DOI:** 10.3897/zookeys.1287.194544

**Published:** 2026-08-03

**Authors:** Rodrigo B. Salvador, Laura Ferreira-Santos, Daniel C. Cavallari, Jonas E. Gallão, Maria E. Bichuette

**Affiliations:** 1 Zoology Unit, Finnish Museum of Natural History, University of Helsinki, Helsinki, Finland Laboratório de Estudos Subterrâneos, Departamento de Ecologia e Biologia Evolutiva, Universidade Federal de São Carlos São Carlos Brazil; 2 Laboratório de Estudos Subterrâneos, Departamento de Ecologia e Biologia Evolutiva, Universidade Federal de São Carlos, São Carlos, SP, Brazil Centro para Documentação da Biodiversidade, Departamento de Biologia, Faculdade de Filosofia, Ciências e Letras de Ribeirão Preto, Universidade de São Paul Ribeirão Preto Brazil; 3 Programa de Pós-Graduação em Biologia Comparada, Departamento de Biologia, Faculdade de Filosofia, Ciências e Letras de Ribeirão Preto, Ribeirão Preto, SP, Brazil Zoology Unit, Finnish Museum of Natural History, University of Helsinki Helsinki Finland; 4 Centro para Documentação da Biodiversidade, Departamento de Biologia, Faculdade de Filosofia, Ciências e Letras de Ribeirão Preto, Universidade de São Paulo, Ribeirão Preto, SP, Brazil Programa de Pós-Graduação em Biologia Comparada, Departamento de Biologia, Faculdade de Filosofia, Ciências e Letras de Ribeirão Preto Ribeirão Preto Brazil; 5 Instituto Brasileiro de Estudos Subterrâneos, São Carlos, SP, Brazil Instituto Brasileiro de Estudos Subterrâneos São Carlos Brazil

**Keywords:** Achatinidae, Amazon Forest, Pará, Scolodontidae, subterranean fauna, Achatinidae, Amazônia, fauna subterrânea, Pará, Scolodontidae, Stylommatophora

## Abstract

The terrestrial gastropod fauna of Brazil is poorly studied, and cave gastropods remain largely unknown in the region. This study provides an initial assessment at the cave-dwelling gastropods of Serra dos Carajás, Pará state, northern Brazil, based on three years of research expeditions to the area. Of the 31 caves explored, 15 contained gastropods. In total, seven species were recorded, four Achatinidae and three Scolodontidae, including one new species in each family (described herein): *Rectobelus arsayae***sp. nov**.; *Dysopeas* sp.; *Leptinaria parana* Pilsbry, 1926; *Leptinaria unilamellata* (d’Orbigny, 1838); *Scolodonta carajaensis***sp. nov**.; *Systrophia siolii* Haas, 1955; *Tamayoa decolorata* (Drouët, 1859). *Systrophia siolii*, however, occurs only in the epigean forested areas, not inside the caves. This study provides the first record of modern specimens of *Rectobelus* in Brazil, the first record of *Tamayoa decolorata* in Brazil, and major range extensions (>500 km) of *Leptinaria parana* and *Systrophia siolii* in Pará, both of which are endemic to this state. Finally, *Nannobeliscus* Weyrauch, 1967 is here considered a synonym of *Rectobelus* Baker, 1927, resulting in the new combination *Rectobelus silvaevagus* (Weyrauch, 1964), **comb. nov**.; and *Nannobeliscus mariaisabelae* Miquel & Jaime, 2018 is considered a junior synonym of *Rectobelus birabeni* (Hylton Scott, 1946).

## Abstract (es)

A fauna de gastrópodes terrestres do Brasil é pouco estudada, e os gastrópodes cavernícolas, em particular, permanecem desconhecidos na região. Este estudo oferece um primeiro olhar sobre os gastrópodes que habitam cavernas na Serra dos Carajás, estado do Pará, norte do Brasil, resultado de três anos de expedições científicas à área. Das 31 cavernas exploradas, 15 apresentaram gastrópodes. Ao todo, foram encontradas sete espécies, sendo quatro da família Achatinidae e três da família Scolodontidae, incluindo uma espécie nova em cada família (descritas neste trabalho): *Rectobelus arsayae***sp. nov**.; *Dysopeas* sp.; *Leptinaria parana* Pilsbry, 1926; *Leptinaria unilamellata* (d’Orbigny, 1838); *Scolodonta carajaensis***sp. nov**.; *Systrophia siolii* Haas, 1955; *Tamayoa decolorata* (Drouët, 1859). No entanto, *Systrophia siolii* ocorre apenas em áreas florestadas epígeas, não dentro das cavernas. Este estudo apresenta o primeiro registro de espécimes modernos de *Rectobelus* no Brasil, o primeiro registro de *Tamayoa decolorata* no país e ampliações significativas de distribuição geográfica (>500 km) de *Leptinaria parana* e *Systrophia siolii* no Pará, sendo ambas espécies endêmicas do estado. Adicionalmente, *Nannobeliscus* Weyrauch, 1967 é aqui considerado um sinônimo de *Rectobelus* Baker, 1927, resultando na nova combinação *Rectobelus silvaevagus* (Weyrauch, 1964), **comb. nov**.; e *Nannobeliscus mariaisabelae* Miquel & Jaime, 2018 é considerado sinônimo júnior de *Rectobelus birabeni* (Hylton Scott, 1946).

## Introduction

The Carajás region, located in southeastern Pará state within the Tocantins River basin in northern Brazil, constitutes one of the most important mineral provinces in the world. The area encompasses the federally protected areas of the Floresta Nacional de Carajás, covering approximately 410,000 ha, and the Parque Nacional dos Campos Ferruginosos, covering ca 80,000 ha, as well as their respective buffer zones. Geologically, the landscape is dominated by ferruginous formations in which hundreds of natural caves have developed in iron-rich rocks, characterizing the Carajás Speleological Province, one of the most significant in Brazil, with more than 1,500 registered subterranean cavities (Lobato et al. 2005; Piló et al. 2015; CANIE 2020). Due to the high economic importance of its mineral resources, the Floresta Nacional de Carajás allows the managed use of natural resources, including mineral exploitation, and currently hosts the largest iron ore mine in the world, in addition to manganese, gold, and granite mining operations (ICMBio 2024), making the region a priority area for biodiversity surveys, environmental assessments, and conservation-oriented studies, particularly regarding subterranean ecosystems.

Caves are well known for harbouring endemic species, often with unique morphological features and biology, and this has been well documented for invertebrate faunas in Brazil (Trajano and Bichuette 2010; Trajano et al. 2016). Gastropods, both terrestrial and freshwater, only partially follow this trend; while a few species are recognised as troglophilic or troglobitic, many species are widespread in surface environments but also occur in caves (Salvador et al. 2022). In general, the study of cave gastropods in Brazil is still in its initial stages of documentation (Salvador et al. 2022), and the northern region of the country, in particular, remains largely unstudied (Salvador 2019). Furthermore, a greater focus has been placed, understandably, on calcareous caves, given the close relationship between snails and calcium carbonate. Ferruginous caves such as the ones in Carajás remain largely unexplored.

The present study represents an initial effort to address this issue. Here we report our findings from cave surveys in the Serra dos Carajás region (Pará state, northern Brazil), focusing on terrestrial gastropods.

## Materials and methods

### Study area and collection

**Figure 1.**
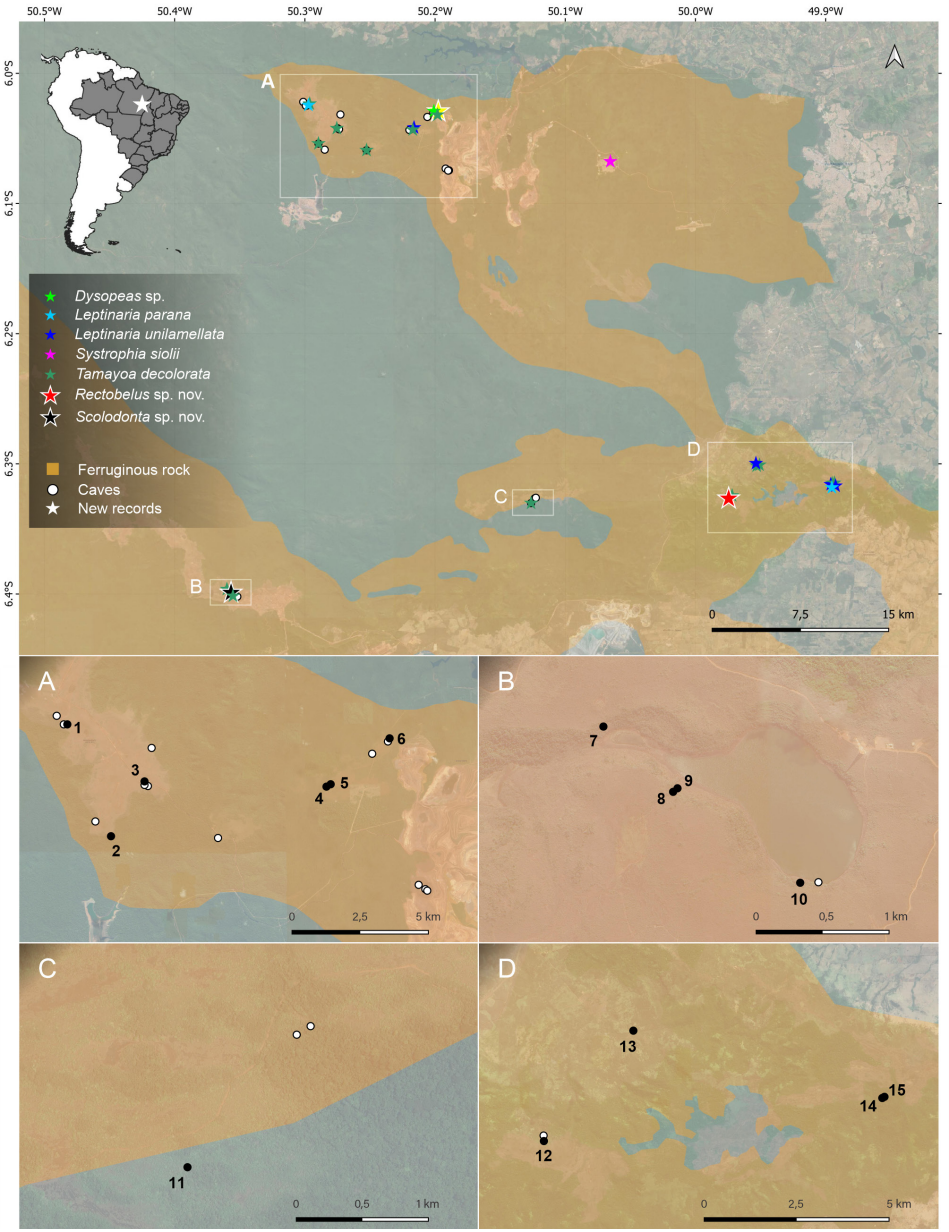
Map of the study region in Pará state, Brazil, showing all visited caves. The general map shows the species found in each cave. Insets (**A–D**) provide a more detailed view of the cave distribution in each area; caves with gastropod occurrences are indicated in black and numbered as follows: (1) N1_0174 (GEM 1374), (2) N1_0208 (GEM 1409), (3) N1_0172 (GEM 1372), (4) N3_0023 (GEM 1911), (5) N3_0026 (GEM 1914), (6) N3_003 (GEM 1873), (7) S11D_0012 (GEM 0653), (8) S11D_0010 (GEM 0651), (9) S11D_0001 (GEM 00650), (10) SD_0013 (GEM 0654), (11) ST_0016, (12) SB_0022 (GEM 1442), (13) SB_0094 (GEM 1510), (14) SB_0049 (GEM 1465, Caverna Samuel 2), (15) SB_0051 (Caverna Samuel 1).

Collection trips to Serra dos Carajás took place in August 2021, February and August 2022, and April 2023. A total of 31 caves were explored in the region, located within two federally protected areas (Floresta Nacional de Carajás also known as FLONA de Carajás, and Parque Nacional dos Campos Ferruginosos), within the municipalities of Canaã dos Carajás and Parauapebas (Fig. 1). The region is part of the Carajás Mineral Province, one of the most important speleological regions in Brazil, where many caves develop in iron formations. The caves surveyed occur in ironstone outcrops locally known as ‘canga’, which form plateaus and ridges across the landscape (Lobato et al. 2005; Piló et al. 2015; CANIE 2020).

Natural cover within the protected areas comprises a mosaic of dense and open tropical forests, deciduous forests, and open xerophilous vegetation associated with ironstone substrates (canga vegetation), which cover the tops of rocky outcrops (Martins et al. 2012). The surroundings of these protected areas, however, have been heavily impacted by deforestation associated with cattle ranching (Martins et al. 2012). The regional climate is strongly seasonal, with a rainy season extending from November to April and a dry season from June to September (Gumier-Costa and Sperber 2009).

Of the 31 caves visited during this study, 15 contained gastropods (Fig. 1). The names (typically codes, e.g., in the formats S11D_0001 and/or GEM 00650) of each cave are indicated below, arranged by the municipality, and include the coordinates and altitude of each cave entrance. **Canaã dos Carajás municipality**: cave S11D_0001 (GEM 00650): 06°23.925'S, 50°21.433'W, 763 m a.s.l.; cave S11D_0010 (GEM 00651): 06°23.932'S, 50°21.442'W, 775 m a.s.l.; cave S11D_0012 (GEM 0653): 06°23.790'S, 50°21.594'W, 755 m a.s.l.; cave SB_0049 (GEM 1465, Caverna Samuel 2): 06°18'58.4"S, 49°53'41.1"W, 629 m a.s.l.; cave SB_0022 (GEM 1442): 06°19.575'S, 49°58.416'W; cave SB_0051 (Caverna Samuel 1): 06°18'57.8"S, 49°53'40.1"W, 638 m a.s.l.; cave SD_0013 (GEM 0654): 06°24'07.9"S, 50°21'09.9"W, 761 m a.s.l.; cave SB_0094 (GEM 1510): 06°18.033'S, 49°57.166'W, 254 m a.s.l.; cave ST_0016: 06°19.802'S, 50°07.583'W, 730 m a.s.l. **Parauapebas municipality**: cave N1_0172 (GEM 1372): 06°02.531'S, 50°16.495'W, 720 m a.s.l.; N1_0174: 06°01'27.20"S, 50°17'54.73"W, 649 m a.s.l.; cave N1_0208 (GEM 1409): 06°03'13.89"S, 50°17'21.59"W, 658 m.s.l.; cave N3_0003 (GEM 1873): 06°02'36.1"S, 50°13'11.6"W, 627 m a.s.l.; cave N3_0023 (GEM 1911): 06°02'36.1"S, 50°13'11.6"W, 627 m a.s.l.; cave N3_0026 (GEM 1914): 06°02'40.3"S, 50°13'10.6"W, 652 m a.s.l.

**Figure 2.**
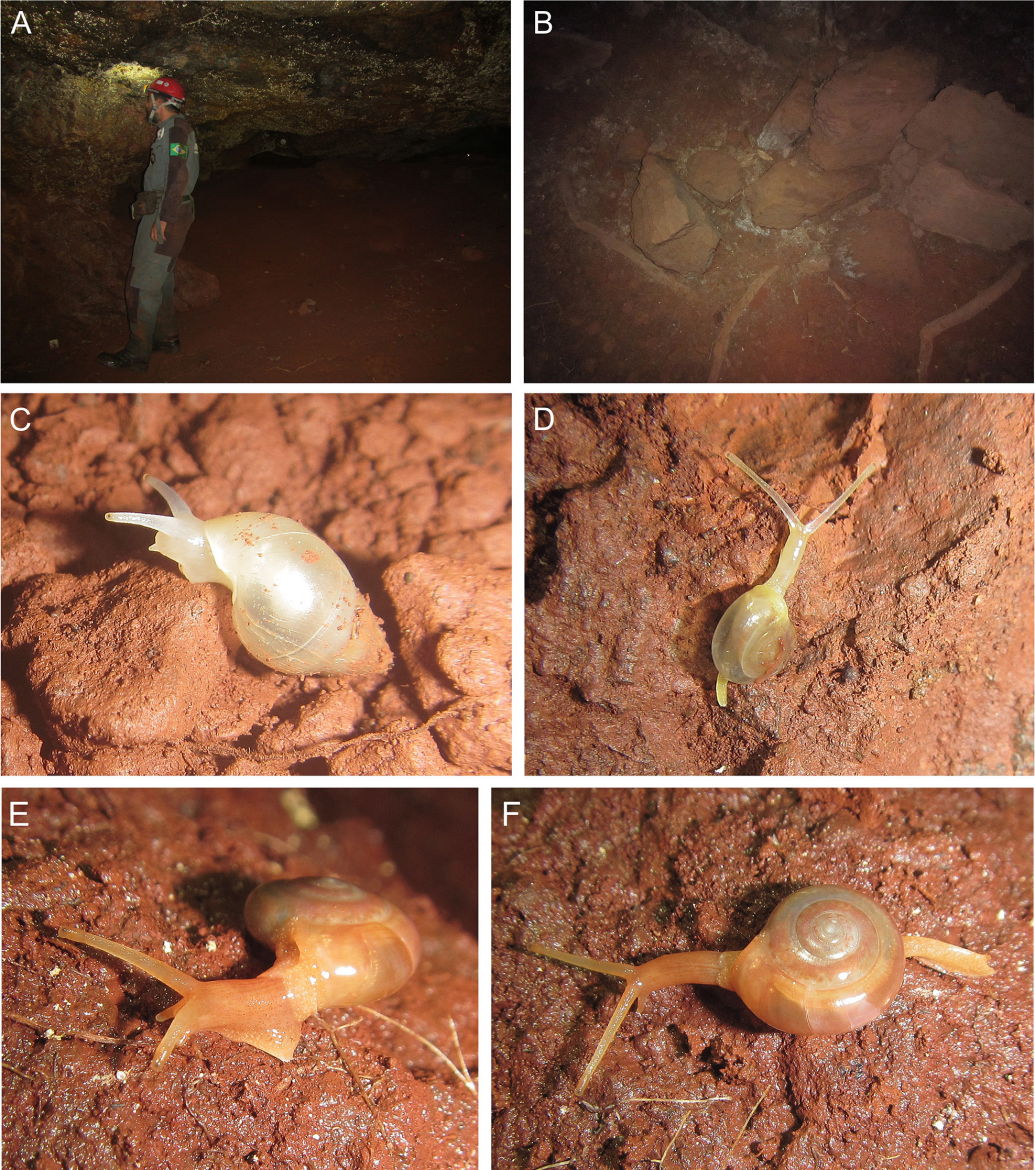
**A, B**. Photographs exemplifying collection localities in the caves. **A**. Interior of cave SB_0022 (GEM 1442), type locality of *Rectobelus arsayae* sp. nov.; **B**. Interior of cave S11D_0001 (GEM 00650), type locality of *Scolodonta carajaensis* sp. nov., showing the type of microhabitat where the holotype of the species was found; **C–F**. Live snails photographed *in situ*. For a precise scale of the specimens, please refer to the text and to Figs 3 and 4; **C**. *Leptinaria* sp., from cave SB_0049 (GEM 1465, Caverna Samuel 2); **D**. *Tamayoa decolorata* (Drouët, 1859), from cave N3_0003 (GEM 1873); **E, F**. *Scolodonta carajaensis* sp. nov., holotype.

In each cave, we conducted visual searches of exposed surfaces and sediment, including rock walls, leaf litter, decaying logs, bat guano deposits, and areas beneath rocks (Fig. 2). Sediment and organic substrates were examined manually, and leaf-litter samples were processed using a Winkler extractor to obtain small invertebrates associated with organic matter. Despite the presence of small water bodies in some caves, no freshwater gastropods were recorded during the survey.

Specimens were preserved either dry (empty shells) or in 70–96% ethanol (live-collected animals) and deposited in the collections of the Laboratório de Estudos Subterrâneos (**LES**), Federal University of São Carlos (**UFSCar**, São Carlos, São Paulo, Brazil), and Coleção Malacológica de Ribeirão Preto (**CMRP**), Faculdade de Filosofia, Ciências e Letras (**FFCLRP**, University of São Paulo, Ribeirão Preto, São Paulo, Brazil). Registration numbers are provided below in the Taxonomy section, under each species entry.

### Imaging

Detailed photographs of the specimens were obtained using a Prime Life Science Cam Intervision 12 MP digital camera mounted on a Nikon SMZ800N stereomicroscope. Image acquisition was performed using PrimeCam Pro software (Prime Instruments, USA). Additional images were captured with a Nikon Coolpix P520 digital camera mounted on a copy stand. Image stacking was carried out using CombineZP software (v. 1.0; Hadley 2010). Final image editing was performed using GIMP (v. 2.8; GIMP Development Team 2012). These procedures enhanced the visibility of morphological characters and facilitated accurate examination and documentation of the specimens.

Computed tomography (CT) scanning was conducted at the Centro para Documentação da Biodiversidade (CDB) using a Phoenix v|tome|x S240 CT & X-Ray System (General Electric, USA) equipped with a digital high-contrast detector (DXR250RT) and a 180 kV nanofocus source, under the following parameters: source voltage of 60–70 kV and current of 200–240 μA, 1,000–1,500 projections; binning of 1×1; averaging three frames with one frame skipped; exposure time of 200.0–333.0 ms; default offset and gain correction; and no filter. Three-dimensional reconstructions were generated using GE’s phoenix datos|x 2.2 software, while visualisation and editing of the 3D models were performed using VGStudio Max 3.0 (Volume Graphics, Germany).

### Molecular data

One specimen per species (for which fresh material was available) was selected for DNA sequencing, including genetic barcoding and other informative and widely used markers in phylogenetic and phylogeographic studies. For the new *Scolodonta* species, we sequenced the holotype and one paratype collected from the other municipality, away from the type locality. A tissue sample from the foot of each scolodontid snail was used for DNA extraction, whereas juvenile specimens of the achatinid snails were used whole.

DNA extraction followed the standard protocol of the QIAGEN DNeasy® Blood & Tissue Kit, including an addition of the final step to increase yield; in this step, one quarter of the buffer volume recommended in the standard protocol was used to avoid excessive dilution of the DNA. We targeted the following markers: (1) the barcoding region of the COI marker using the standard invertebrate primers LCO1490/HCO2198 (Folmer et al. 1994); (2) the mitochondrial 16S marker, employing the primers 16SarL/16SbrH (Simon et al. 1994); and (3) a contiguous sequence of nuclear DNA containing the 3' end of the 5.8S rRNA gene, the entire ITS2 region, and a large portion of the 5' end of the 28S rRNA gene, amplified in 2 fragments using primers LSU-1/LSU-3 and LSU-2/LSU-5 (Wade and Mordan 2000; Wade et al. 2007).

PCR amplification of the COI and 16S markers consisted of an initial denaturation of 3 m at 96 °C; followed by 35 cycles of 30 s denaturation at 95 °C, 1 min annealing at 48 °C (up to 51 °C for 16S), and 2 min extension at 72 °C; and a final 5 min extension at 72 °C. Amplification of the ITS2+28S markers differed in the cycles, consisting of 40 cycles of 30 s denaturation at 95 °C, 1 min annealing at either 50 °C (ITS2 section) or 45 °C (28S section), and 2 min extension at 72 °C. PCR success was assessed by agarose gel electrophoresis, and PCR products were purified using ExoSAP-IT™ (Affymetrix Inc.). Samples were prepared for Sanger sequencing, which was conducted externally at Macrogen Europe (Amsterdam, The Netherlands).

The resulting sequences were de novo assembled and quality-checked in Geneious Prime (v.2025, Biomatters Ltd.). Consensus sequences were uploaded to GenBank; accession numbers are provided under each species entry in the Taxonomy section.

## Taxonomy

### Superfamily Achatinoidea Swainson, 1840

**Family Achatinidae Swainson, 1840**

**Subfamily Stenogyrinae Fischer & Crosse, 1877**

#### Genus Rectobelus

Taxon classificationAnimaliaStylommatophoraAchatinidae

Baker, 1927

8C1EC4F4-07DE-567E-B170-FFCBCBD679B7

Obeliscus (Rectobelus) Baker, 1927: 6.
Obeliscus (Microbeliscus) Weyrauch, 1964: 40 [type species, by original designation: Obeliscus (Microbeliscus) silvaevagus Weyrauch, 1964].
Obeliscus (Nannobeliscus) Weyrauch, 1967: 458 [new name for Obeliscus (Microbeliscus) Weyrauch, 1964 non Sandberger, 1875].

##### Type species.

Obeliscus (Rectobelus) rectus Baker, 1927 [Venezuela, La Fría], by original designation.

##### Included species.

*Rectobelus birabeni* (Hylton Scott, 1946) [=*Nannobeliscus mariaisabelae* Miquel & Jaime, 2018, syn. nov.], *Rectobelus levogyrus* Simone & D’ávila, 2019, *Rectobelus rectus* Baker, 1927, *Rectobelus silvaevagus* (Weyrauch, 1964), comb. nov.

##### Diagnosis.

Small shell (~ 10–15 mm), multispiral (8–12 whorls), elongated and very narrow (conico-elongated to nearly cylindrical). Protoconch bulbous. Shell smooth, except for growth lines. Aperture quadrangular, with simple peristome. Weak columellar fold may be present in adults. Internal palatal lamellae may be present.

##### Distribution.

Venezuela (Baker 1927), northern Brazil (Pará state; this study), central Peru (Weyrauch 1964, 1967), and northern Argentina (Jujuy province; Hylton Scott 1946; Miquel and Jaime 2018). It is also known from Rondônia state, western Brazil; however, this record is based exclusively on shells from archaeological contexts (shell mounds dated ca 4500 BP), and it is currently unclear whether the species is still extant (Simone and D’ávila 2019).

##### Remarks.

*Rectobelus* has been described as a “section” of *Obeliscus* Beck, 1837 and has been since recognised as either its subgenus or synonym (Baker 1927; Zilch 1960), or as a synonym of *Ischnocion* Pilsbry, 1907 (Hausdorf et al. 2012). Alternatively, it has been considered a genus on its own (Schileyko 1999), a classification recently supported by the revision of Simone and D’ávila (2019). Similarly, *Nannobeliscus* has been deemed a subgenus of *Obeliscus* (Weyrauch 1964, 1967), a synonym of *Ischnocion* together with *Rectobelus* (Hausdorf et al. 2012), or a separate genus (Schileyko 1999; Miquel and Jaime 2018). The species of both genera are extremely similar in shell shape and size, the known anatomical differences are minimal (see below), and the distributions of both genera are consistent with one another, including a co-occurrence at the same locality in northern Argentina. Thus, herein we reinforce that *Nannobeliscus* is a synonym of *Rectobelus* (cf. Hausdorf et al. 2012). That results in the new combination of the type species of *Nannobeliscus* as *Rectobelus silvaevagus* (Weyrauch, 1964), comb. nov.

*Rectobelus birabeni* and *Nannobeliscus mariaisabelae* were described from the same province in Argentina and have indistinguishable shells, exhibiting the same shape, size (~ 14.5–16.0 mm), and number of whorls (11–12), as well as having identical radulae (Hylton Scott 1946; Miquel and Jaime 2018; Simone and D’ávila 2019). The only conchological differences reported were the presence of a palatal lamella in some juveniles of the former and a weak columellar fold in adults of the latter (Miquel and Jaime 2018), neither of which is supported here. Firstly, as described by Hylton Scott (1946) based on a series of juvenile and adult specimens, and reinforced here through CT-scan imaging (see below), the palatal lamellae are present at certain stages during the animal’s growth. As the snail grows, the lamellae become internalized in the shell and are, therefore not visible in adults. Secondly, the columellar fold was also reported by Hylton Scott (1946) in some specimens of *R. birabeni*. As argued by Simone and D’ávila (2019), the columellar fold is variable within many species of South American achatinids and is not particularly reliable for diagnosis by itself. Finally, the subtle anatomical differences noted by Miquel and Jaime (2018) in the size and shape of the penis and spermatheca duct are very tenuous and known to vary intraspecifically, as well as seasonally, in stylommatophorans (Baur and Baur 2017). As such, *Nannobeliscus mariaisabelae* should be considered a junior synonym of *R. birabeni*.

#### Rectobelus arsayae sp. nov.

Taxon classificationAnimaliaStylommatophoraAchatinidae

C201E9A6-AF1D-561E-9426-335EF2F585C6

https://zoobank.org/4574D42B-51A7-4EA3-AA68-6CF0395377A9

Fig. 3A–C

##### Type material.

***Holotype***LES 0031661.

##### Type locality.

Brazil, Pará state, Canaã dos Carajás municipality, cave SB_0022 (GEM 1442) (JE Gallão & DB Ribeiro leg., 06.iv.2023).

##### Diagnosis.

Shell nearly cylindrical in shape, with ca 10 whorls and ca 10 mm in length. The apex is not as narrow as in congeners and the protoconch is only slightly inflated relative to the first teleoconch whorls. Presence of very faint columellar fold, extending internally into the shell throughout the length of the body whorl. Presence of two internal palatal lamellae in sequence; the external-most one is ~ ¹/3 whorl deep into the shell, and the second one is ~ ¹/2 whorl in distance from the first one.

**Figure 3.**
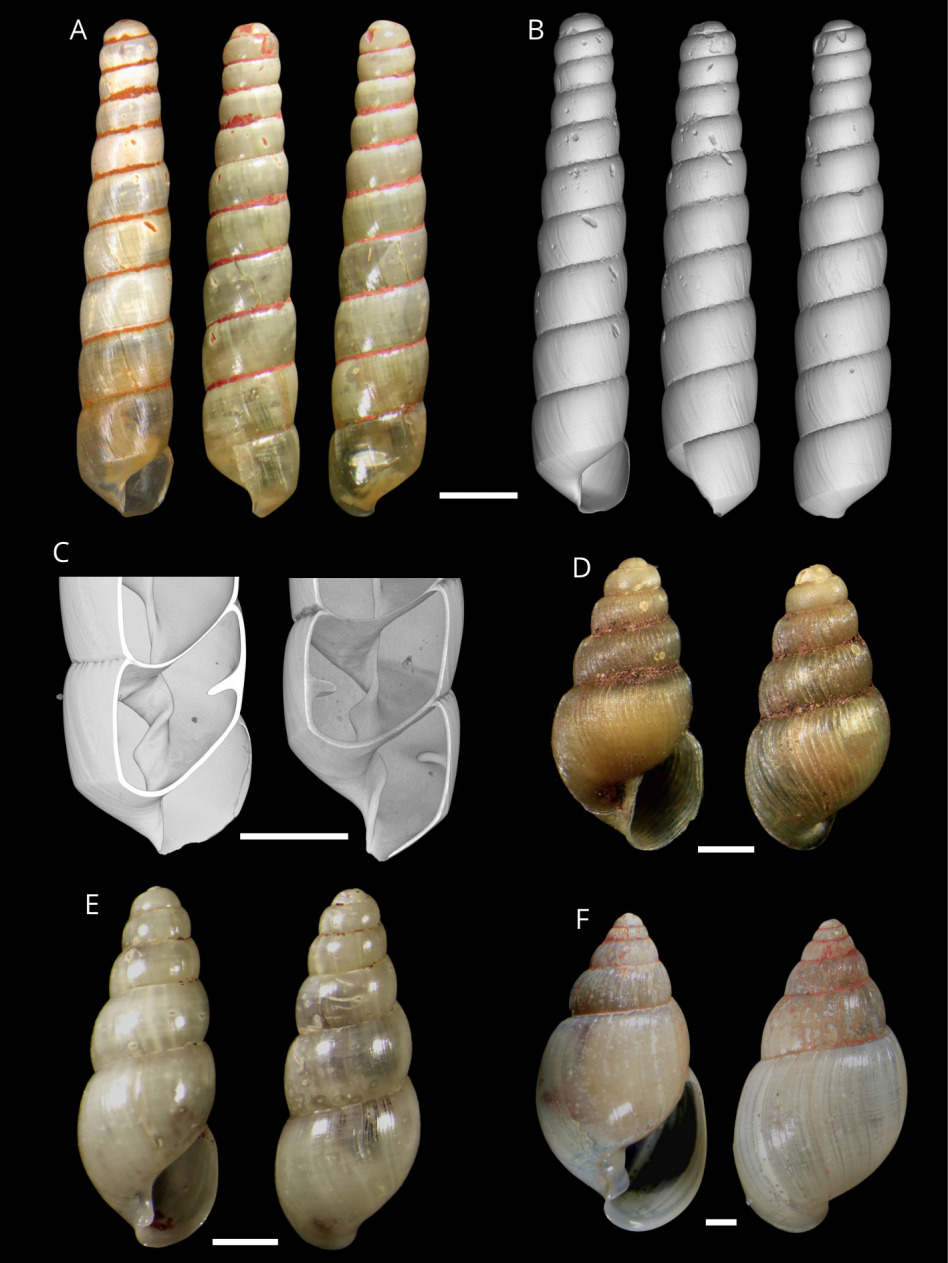
Representatives of family Achatinidae. **A, B**. *Rectobelus arsayae* sp. nov., holotype (LES 31661): **A**. stereomicroscope images; **B**. 3D reconstruction from micro-CT data (voxel size = 11.75 µm). **A, B**. View of entire shell in apertural, lateral, and dorsal views, respectively; **C**. Internal shell structures, showing faint columellar fold and two internal palatal lamellae; **D**. *Dysopeas* sp. (LES 0031639); **E**. *Leptinaria parana* (LES 0031622); **F**. *Leptinaria unilamellata* (LES 0031650). Scale bars: 1 mm.

##### Description.

Shell small (shell height = 10.2 mm; shell width = 1.9 mm), multispiral (10 ¹/2 whorls), narrow, elongated, nearly cylindrical. Shell colour whitish yellow to light ochre. Protoconch (~ 2 whorls) bulbous, smooth. Teleoconch smooth, except for prosocline growth lines. Whorls increase regularly in height and only slightly in width. Suture well-marked, but not deep. Aperture quadrangular. Peristome simple. Weak columellar fold present on body whorl. Two internal palatal lamellae present on the body and penultimate whorl, spaced ~ ¹/2 whorl apart.

##### Distribution.

Known only from the type locality.

##### Habitat.

Found in the twilight zone of the cave, under a rock. The specimen is likely autochthonous, given that the fragility of the shell would not allow extensive transport.

##### Etymology.

Considering that the type locality is a cave in Canaã dos Carajás, the specific epithet honours Arsay, the Canaanite (Ugaritic) goddess of the underworld (an equivalent to the Hurrian Allani and the Mesopotamian Ereshkigal).

##### Remarks.

Only a single shell of this species was found during the study, despite extensive searches. Nevertheless, it is biogeographically highly isolated (both in physical distance and in biome) and exhibits a distinct different morphology from other species in the genus, allowing us to confident assign it to a new species. *Rectobelus arsayae* sp. nov. can be readily diagnosed from its congeners by its more cylindrical shell, whereas the other species exhibit more conical shell profiles. As the spire of *R. arsayae* sp. nov. is broader compared e to the more tapering spire of its congeners, its protoconch seems less inflated. Its shell is larger than that of the Peruvian *R. silvaevagus* comb. nov. (~ 8.5 mm shell height and 9 ¹/2 whorls) and much smaller than the Argentine *R. birabeni*, including the newly synonymized *R. mariaisabelae* comb. nov. (~ 14.5–16.0 mm shell height and 11–12 whorls). It is of similar size to the Venezuelan *R. rectus* (~ 9.0–10.5 mm shell height and 10–11 whorls) and the sinistrally-shelled Brazilian subfossil *R. levogyrus* (~ 10.5–11.0 mm, shell height and 8–10 whorls).

The shell of *Rectobelus arsayae* sp. nov. also exhibits a very faint columellar fold, which extends internally along the entire length of the body whorl, as visible in the tomography images (Fig. 3C). That feature was previously known only in *R. birabeni* but, as explained above, this could be a variable character. Furthermore, tomography images revealed the presence of two internal palatal lamellae that appear in sequentially during shell growth (Fig. 3C). The outermost lamella is ~ ¹/3 whorl deep into the shell from the aperture, and the second one is ~ ¹/2 whorl in distance from the first. Again, a similar feature has only been reported in *R. birabeni*, but other species have not been examined for juveniles or using tomography in the adult shell.

The present record greatly extends the known distribution of the genus in South America and also represents the first report of modern *Rectobelus* in Brazil (*R. levogyrus* is known from archaeological settings only; Simone and D’ávila 2019).

### Subfamily Subulininae Fischer & Crosse, 1877

#### Genus *Dysopeas* Baker, 1927

##### Dysopeas

Taxon classificationAnimaliaStylommatophoraAchatinidae

sp.

2639597B-9E86-566A-9E2E-D51A73547C94

Fig. 3D

###### Material examined.

LES 0031639, LES 0031637, LES 0031641 (Parauapebas, cave N3_0003; JE Gallão, TS Gallo, DF Torres & JEM Santos leg., 13.viii.2022).

###### GenBank acc. nr.

PZ576039 (COI), PZ574756 (16S), PZ577561 (ITS2+28S) (LES 0031639).

###### Identification.

The shells of the present specimens are consistent with the genus *Dysopeas*, distributed from Nicaragua to southeastern Brazil (Simone 2006; López et al. 2015). In particular, although faint, the protoconch sculpture supports assignment to this genus: axial riblets on the apical-most area of the whorl (near the suture) and spiral lines on the remainder of the shell surface, continuing into the transition to the teleoconch after the axial teleoconch ribbing begins (Baker 1927; López et al. 2015). The overall shell shape and ribbing pattern do not fully match those of other species in Brazil and Venezuela; thus, the present specimens may represent a new species. However, considering that the specimens are juveniles, this cannot be confirmed at present.

Notably, the COI marker of the present specimen shows only 80.0–81.5% identity with the available sequences of *Dysopeas muibum* (Marcus & Marcus, 1968) from Rio de Janeiro (Fernandes et al. 2026), which is currently the only member of the genus recorded from Brazil (Salvador et al. 2024).

###### Remarks.

The specimens were found in the entrance and twilight zones of a single cave, located either under rocks on unconsolidated humid substrate or on guano of frugivorous and nectarivorous bats.

#### Genus *Leptinaria* Beck, 1837

##### Leptinaria parana

Taxon classificationAnimaliaStylommatophoraAchatinidae

Pilsbry, 1926

E19CB8B3-C12D-5A6C-A61B-60EF5990BF18

Fig. 3E

Leptinaria parana : Simone and Martins 2020: 19, fig. 5 [see this article for full synonymy]; Salvador et al. 2024: 151.

###### Material examined.

LES 0031622, LES 0031653 (Canaã dos Carajás, cave SB_0049; JE Gallão, JS Gallo, DF Torres & VF Sperandei leg., 16.ii.2022) • LES 0031655 (Parauapebas, cave N1_0174; JE Gallão & DB Ribeiro leg., 04.iv.2023).

###### GenBank acc. nr.

PZ576040 (COI), PZ574757 (16S), PZ577562 (ITS2+28S) (LES 0031622).

###### Identification.

This species can be identified by its small size (~ 5.5 mm height, ~ 2 mm width, ~ 6 whorls), elongated spire, and faint growth lines (Pilsbry 1926).

###### Distribution.

Known only from the type locality in Pará (Pilsbry 1906), interpreted as a suburb of Belém (see discussion in Simone and Martins 2020). Records of *L. parana* beyond the type locality are likely incorrect. Based on the image provided in their work, the occurrence of *L. parana* in Peru reported by Ituarte et al. (2008) likely corresponds to a different species. The record of *L. parana* from the state of Santa Catarina, southern Brazil, reported by Agudo-Padrón (2018) and follow-up works (Agudo-Padrón 2022, 2024), with authorship erroneously attributed to “Pilbry, 1906”, is likewise dubious. The vouchers could not be traced; the illustration provided by Agudo-Padrón (2018: 58, fig. 2) is inconclusive (showing only a dorsal view of the shell) and likely corresponds to other congeners known from that area (see Simone 2006). The present record extends the species’ range by ca 550 km south of its previous record in Belém.

###### Remarks.

In the studied caves, *L. parana* was found in the twilight zone (in leaf litter) and the aphotic zone (under rocks, on very humid unconsolidated substrate). Furthermore, the species has also been recorded in anthropically disturbed areas in the nearby municipality of Parauapebas (Paulo Ricardo S. Coelho, pers. comm. 23 March 2026, species identity confirmed via photographs).

##### Leptinaria unilamellata

Taxon classificationAnimaliaStylommatophoraAchatinidae

(d’Orbigny, 1838)

4AA0A776-7D96-55D3-AA8D-877084AC4266

Fig. 3F

Leptinaria lamellata : Morretes 1959: 132; Salgado and Coelho 2003: 155; Simone 2006: 186, fig. 678; Salvador et al. 2021: 5.
Leptinaria unilamelata [sic]: Oliveira et al. 1981: 327.
Leptinaria unilamellata : Simone 2006: 186, fig. 684; Breure et al. 2016: 35, tab. 3, fig. 14; Alexandre et al. 2017: 34, tabs 1–2, fig. 2K; Breure et al. 2020: 27, figs 1–5 [see this article for full synonymy]; Lima et al. 2021: 274, fig. 3T–V; Rangel et al. 2021: 4, tab. 1, fig. 2b; Silva et al. 2021: 48, fig. 4G, H; Salvador et al. 2024: 151.

###### Material examined.

LES 0031620, LES 0031621, LES 0031623 (Canaã dos Carajás, cave SB_0049; JE Gallão, JS Gallo, DF Torres & VF Sperandei leg., 16.ii.2022) • LES 0031629 (Parauapebas, cave N3_0026; JE Gallão, JS Gallo, DF Torres & VF Sperandei leg., 18.ii.2022) • LES 0031643 (Parauapebas, cave N3_0026; JE Gallão, TS Gallo, DF Torres & JEM Santos leg., 16.viii.2022) • LES 0031655 (Canaã dos Carajás, cave SB_0049; JE Gallão & DB Ribeiro leg., 04.iv.2023) • LES 0031657 (Canaã dos Carajás, cave SB_0094; JE Gallão & DB Ribeiro leg., 06.iv.2023) • LES 0031648, LES 0031650 (Canaã dos Carajás, cave SB_0049; JE Gallão & DB Ribeiro leg., 12.iv.2023) • LES 0031648 (Canaã dos Carajás, cave SB_0051; JE Gallão & DB Ribeiro leg., 12.iv.2023).

###### GenBank acc. nr.

PZ574924 (16S), PZ577563 (ITS2+28S) (LES 0031650).

###### Identification.

*Leptinaria unilamellata* is a very widespread species in the Neotropics, characterised by the single lamella that gives it its name, and known from both natural and anthropically disturbed areas. It is thought to represent a species complex, but genetic and morphometric studies would be required to clarify that. In any case, the species shows conchological variation, with most shells being smooth, while axially ribbed specimens are uncommon but well known. These two shell morphs are known to occur in the same place in southeastern Brazil (DCC, pers. obs.), and both were found in the present material.

###### Distribution.

Nicaragua to southern Brazil (Simone 2006; Delannoye et al. 2015; Silva et al. 2021).

###### Remarks.

In the studied cave, *Leptinaria unilamellata* was found in the aphotic zone. Individuals were found under rocks on humid unconsolidated substrate, among roots, or on bat guano (mixed guano of frugivorous and insectivorous bats). This species has also been recorded in anthropically disturbed areas in the nearby municipality of Parauapebas in Pará (Paulo Ricardo S. Coelho, pers. comm., species identity confirmed via photographs).

### Superfamily Scolodontoidea Baker, 1925

**Family Scolodontidae Baker, 1925**

#### Genus *Scolodonta* Doering, 1875

##### Scolodonta carajaensis sp. nov.

Taxon classificationAnimaliaStylommatophoraScolodontidae

A35F2D59-EF11-564F-BBDE-A9B084EEB5DB

https://zoobank.org/7368504-A31D-41A9-9EB3-9B621D6BF00D

Figs 2E–F, 4A–C

###### Type material.

***Holotype***LES 0031619. ***Paratypes***LES 0031618 (collected together with holotype) • CMRP 1300 (ex LES 0031640) (Parauapebas, cave N3_0003; JE Gallão, TS Gallo, DF Torres & JEM Santos leg., 13.viii.2022).

###### Type locality.

Brazil, Pará state, Canaã dos Carajás municipality, FLONA de Carajás, Serra Sul, cave S11D_0001 (GEM 00650) (JE Gallão, JS Gallo, FD Torres & VF Sperandei leg., 11.ii.2022).

###### Diagnosis.

Spire elevated; body whorl short, with rounded profile (lacking a marked angulation); aperture D-shaped, rounded; peristome simple.

**Figure 4.**
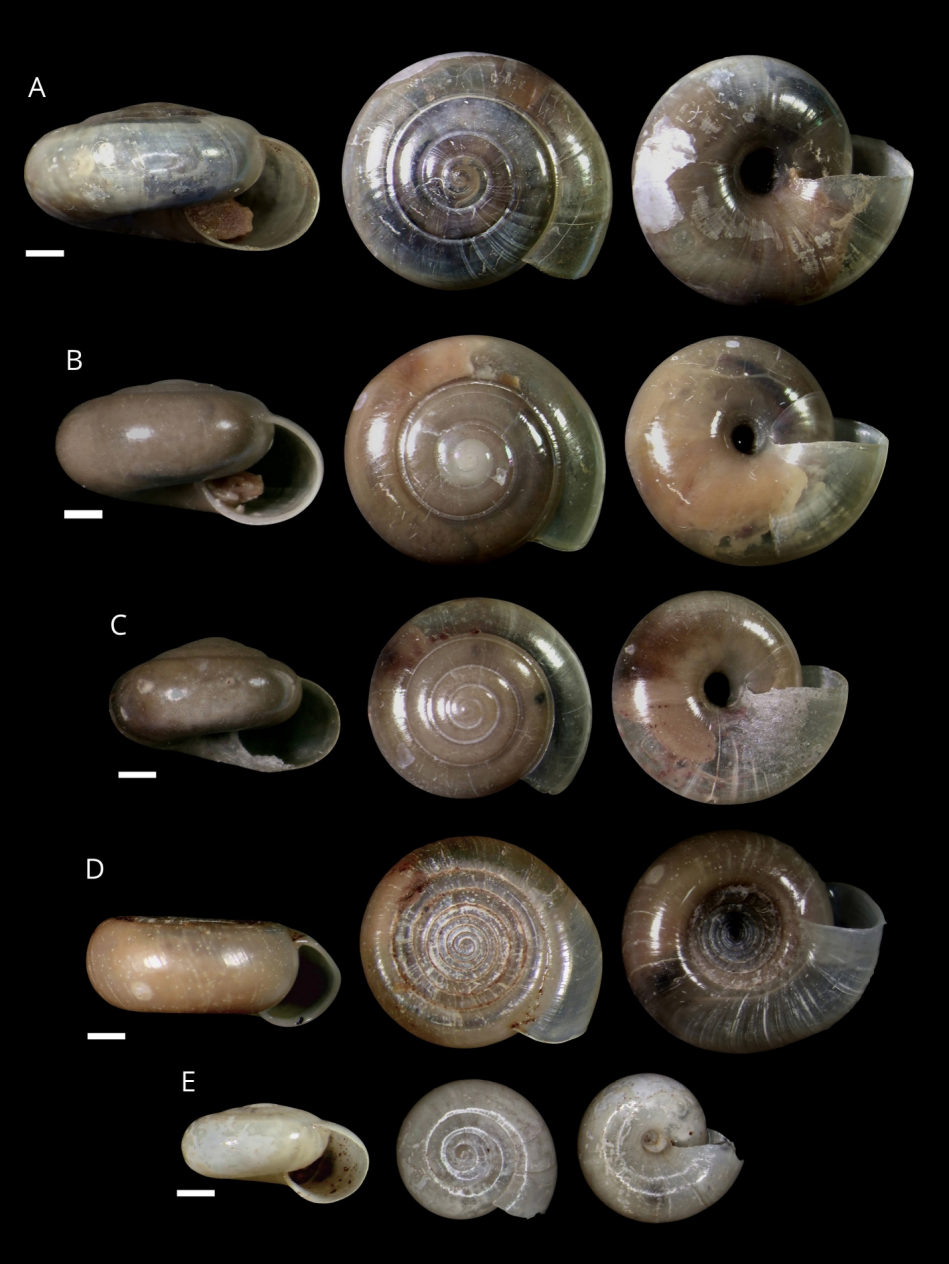
Representatives of family Scolodontidae, shown in apertural, apical and umbilical views. **A–C**. *Scolodonta carajaensis* sp. nov.: **A**. Holotype (LES 0031619); **B**. Paratype (LES 0031618); **C**, paratype (CMRP 1300); **D**. *Systrophia siolii* (LES 0031645); **E**. *Tamayoa decolorata* (LES 0031616). Scale bars: 1 mm.

###### Description.

Shell small (~ 6 mm wide), discoid, with ~ 4 ¹/2 whorls; glossy and translucent, tan in colour. Shell smooth except for fine growth lines. Protoconch seemingly has 1 ^1^/2 whorl, but transition to teleoconch is indistinct. Spire slightly elevated; spire whorls with a weakly convex profile. Suture well marked, slightly deep. Whorls increase regularly in both width and height. Body whorl profile rounded. Aperture D-shaped, rounded. Insertion of aperture on body whorl just 0.1–0.2 mm over the median region of whorl. Peristome simple, sharp. Umbilicus wide, deep. Shell measurements (holotype): 4 ¹/2 whorls, shell height = 3.0 mm, shell width = 6.0 mm.

Soft body, orange-red (Fig. 2E, F); some parts (middle of the eyestalks, tip of lower tentacles, border of the foot, edge of the mantle) are slightly paler and less reddish. Head-foot, marked by longitudinal rows of minute and more darkly pigmented spots. Head-foot narrow, with very narrow “tail”; eyestalks proportionately long (around the same size as frontal end of head-foot). Mantle and soft parts are partially visible through the shell.

###### GenBank acc. nr.

PZ576041 (COI), PZ575549 (16S) (LES 0031619); PZ576042 (COI), PZ575550 (16S), PZ575551 (ITS2+28S) (CMRP 1300).

###### Distribution.

Known from caves in the municipalities of Canaã dos Carajás (type locality) and Parauapebas, in Serra dos Carajás.

###### Habitat.

Found from the entrance to the aphotic zones of the caves. Live individuals were found in very humid microhabitats: under rock, in an area with plenty of organic matter and plant roots (in Canaã dos Carajás), and on unconsolidated substrate close to water drip (in Parauapebas).

###### Etymology.

The specific epithet alludes to the study area, Serra dos Carajás.

###### Remarks.

*Scolodonta* is primarily identified by its glossy and often translucent shell (Hausdorf 2006), which is slightly sturdier-looking than those of other glossy-shelled scolodontids (e.g., *Happia* Bourguignat, 1890, *Systrophiella* Baker, 1925). The lack of shell sculpture (except for fine growth lines) and the weakly elevated spire are also diagnostic of the genus (Hausdorf 2006).

We are confident in assigning our specimens to a new species, even though two species of “*Scolodonta*” are already known from Pará state. Dohrn (1882) described both *Scolodonta nitidula* (Dohrn, 1882) and *Scolodonta amazonica* (Dohrn, 1882) (originally allocated to *Ammonoceras* Pfeiffer, 1855) from the margins of the Amazon River in Pará. While his descriptions are informative, the original study lacks illustrations. Neither species has been figured since, and the type material is likely lost (Dance 1966). Nevertheless, based on the descriptions and discussion in Dohrn (1882), his *S. nitidula* is likely synonymous with *Tamayoa decolorata* (see below), which has a smaller and more rounded shell, as well as a yellow head-foot. On the other hand, *S. amazonica* is described as having a punctiform and barely visible umbilicus (Dohrn 1882), which is immediately inconsistent with *Scolodonta carajaensis* sp. nov. Furthermore, *S. amazonica* has a reflexed peristome around the columella and an even rounder aperture in comparison to *S. nitidula* / *T. decolorata* (Dohrn 1882), which is again inconsistent with the new species.

*Scolodonta carajaensis* sp. nov. can be readily distinguished from other Brazilian Scolodontidae. *Scolodonta bounobaena* (d’Orbigny, 1835) is similar in overall shell shape, but it is slightly smaller, with thicker walls; also, it has a stronger angulation in the median-apical region of the body whorl, and a lightly thickened peristome. *Scolodonta interrupta* (Suter, 1900) is similar in size but has a much more raised spire and a more circular aperture. Furthermore, the periodical interruptions seen in the shell of *S. interrupta* are more reminiscent of members of the family Streptaxidae, which could indicate an incorrect allocation (Salvador 2021). *Scolodonta spirorbis* (Deshayes, 1850) is much smaller, with a fragile discoid shell, and very likely does not belong in *Scolodonta* (Roosen et al., unpub. data).

The occurrence of this species in more than one cave across the two municipalities suggests that the species is more widespread on the surface across Serra dos Carajás. Such an established epigean population is supported by genetic evidence; there is a 100% match of both COI and 16S sequences of the holotype and paratype, one from each municipality, indicating interconnectivity and gene flow. Furthermore, no obvious troglomorphisms were observed; the translucent shell is very common in multiple members of this family (Roosen and Breure 2024a, 2024b; Roosen et al. 2025a, 2025b) and cannot be considered a troglomorphism per se.

#### Genus *Systrophia* Pfeiffer, 1855

##### Systrophia siolii

Taxon classificationAnimaliaStylommatophoraScolodontidae

Haas, 1955

4E758E13-E50C-56F8-8A41-661DB4CC4A04

Fig. 4D

Systrophia (Systrophia) siolii Haas, 1955: 103, figs 4–6; Solem 1967: 129.
Systrophia siolii : Richardson 1989: 137; Salgado and Coelho 2003: 169; Simone 2006: 223, fig. 845; Salvador et al. 2024: 153.

###### Material examined.

LES 0031645 (Canaã dos Carajás, Base ICMBio Gavião Real, Águas Claras; JE Gallão, JS Gallo, DF Torres & JEM Santos leg., viii.2022).

###### Identification.

The discoid, smooth shell (~ 4 mm height, ~ 9.5 mm width, ~ 8 whorls), with tightly coiled whorls and the body whorl suddenly expands near the aperture, is consistent with *Systrophia siolii*, known from elsewhere in Pará state (Simone 2006).

###### Previously known distribution.

Known only from the type locality, Serra Formosa near Mulata, Monte Alegre, Pará (Haas 1955; Simone 2006). This species was found in the woods of the epigean zone near the caves, but was not found inside any caves per se. In any event, the present record extends its range ca 630 km southeast of its previous record in Monte Alegre. Furthermore, the species has also been found in anthropically disturbed areas in the nearby municipality of Parauapebas (Paulo Ricardo S. Coelho, pers. comm. 23 March 2026, species identity confirmed via photographs).

#### Genus *Tamayoa* Baker, 1925

##### Tamayoa decolorata

Taxon classificationAnimaliaStylommatophoraScolodontidae

(Drouët, 1859)

C27DBC1D-CFD9-5FAB-AA8B-6D673D63A3A1

Figs 2D, 4E

Zonites decoloratus Drouët, 1859: 50, pl. 1, figs 3–5.
Helix decolorata : Pfeiffer 1868: 169.
Zonites (Hyalinia) decolorata : Tryon 1886: 166, pl. 52, figs 69–71.
Scolodonta (Happia) decolorata : Kobelt 1905–1906: 68, pl. 51, figs 14, 15.
Scolodonta decolorata : Kobelt 1910: 146.
Happia decolorata : Baker 1925: 23; Richardson 1989: 119.
Tamayoa (Tamayops) decolorata : Tillier 1980: 106, pl.5, fig. 1, figs 86–91.
Tamayoa (Tamayoa) decolorata : Schileyko 2000: 759, fig. 988A–C.
Happiella decolorata : Chase and Robinson 2001: 49; Rosenberg and Muratov 2006: 140, tab. 2.
Tamayoa decolorata : Massemin et al. 2009: 423, text fig., pl. 11, fig. B; Robinson et al. 2009: 640, figs 6F, 9B; Delannoye et al. 2015: 282, pl. 62; Charles 2016: 50, pl. 2, fig. 5; Sei et al. 2017: 705, tab. 2; Salvador 2021: 66; González-Guillén and Teruel 2022: 111; Wagner 2022: 24; Georgiev and Mishev 2025: 4.

###### Material examined.

LES 0031616 (Parauapebas, cave N1_0174; ME Bichuette, JE Gallão & DF Torres leg., 22.viii.2021) • LES 0031624, LES 0031625 (Canaã dos Carajás, cave SB_0049; JE Gallão, JS Gallo, DF Torres & VF Sperandei leg., 16.ii.2022) • LES 0031656 (Parauapebas, cave N1_0172; JE Gallão & DB Ribeiro leg., 05.iv.2023) • LES 0031630 (Parauapebas, cave N1_0174; JE Gallão, JS Gallo, DF Torres & VF Sperandei leg., 12.ii.2022) • LES 0031654 (Parauapebas, cave N1_0174; JE Gallão & DB Ribeiro leg., 04.iv.2023) • LES 0031626, LES 0031627 (Parauapebas, cave N1_0208; JE Gallão, JS Gallo, DF Torres & VF Sperandei leg., 19.ii.2022) • LES 0031638, LES 0031642 (Parauapebas, cave N3_0003; JE Gallão, JS Gallo, DF Torres & JEM Santos leg., 13.viii.2022) • LES 0031617 (Parauapebas, cave N3_0023; ME Bichuette, JE Gallão & DF Torres leg., 25.viii.2021) • LES 0031628 (Parauapebas, cave N3_0026; JE Gallão, JS Gallo, DF Torres & VF Sperandei leg., 18.ii.2022) • LES 0031631 (Parauapebas, cave S11D_0013; JE Gallão, JS Gallo, DF Torres & VF Sperandei leg., 15.ii.2022) • LES 0031659, LES 0031660 (Canaã dos Carajás, cave SB_0022; JE Gallão & DB Ribeiro leg., 06.iv.2023) • LES 0031651, LES 0031652 (Canaã dos Carajás, cave SB_0049; JE Gallão & DB Ribeiro leg., 12.iv.2023) • LES 0031632, LES 0031633, LES 0031634 (Canaã dos Carajás, cave SB_0051; JE Gallão, JS Gallo, DF Torres & VF Sperandei leg., 16.ii.2022) • LES 0031644 (Canaã dos Carajás, cave SB_0051; JE Gallão, JS Gallo, DF Torres & JEM Santos leg., 17.viii.2022) • LES 0031649 (Canaã dos Carajás, cave SB_0051; JE Gallão & DB Ribeiro leg., 12.iv.2023) • LES 0031658 (Canaã dos Carajás, cave SB_0094; JE Gallão & DB Ribeiro leg., 06.iv.2023) • LES 0031646, LES 0031647 (Canaã dos Carajás, cave ST_0016; JE Gallão & DB Ribeiro leg., 13.iv.2023).

###### Identification.

The smooth shell, its size (~ 4 mm height, ~ 7 mm width, ~ 4 whorls), and profile, the slight constriction of the body whorl close to the aperture, and the whitish-yellow body (Fig. 2D), with a distinct yellow tint on the visible mantle area, all support its identification as *Tamayoa decolorata* (Massemin et al. 2009; Delannoye et al. 2015). The present specimens could also fit in the description of *Scolodonta nitidula* (Dohrn, 1882) from the margins of the Amazon River in Pará. However, the original description lacks illustrations, the species has never illustrated since, and the type material is likely lost (Dance 1966). Considering the comparisons made by Dohrn (1882) between his species and *Tamayoa decolorata* and *Hapiella surinamensis* (Pfeiffer, 1872), it is very likely that his *Scolodonta nitidula* is synonymous with *Tamayoa decolorata*.

###### Distribution.

This species is known from French Guiana and is considered to have been introduced to the Antilles: Barbados, Dominica, Guadeloupe, Jamaica, and Martinique (Delannoye et al. 2015). The present record is the first report of this species from Brazil, although it is not the first time a land snail previously known from French Guiana has been recorded in northern Brazil (Salvador et al. 2018, 2020). A previous record of *T. decolorata* from southeastern Brazil (Esteves et al. 2025) has been considered erroneous (Salvador et al. 2026).

###### Remarks.

In the studied caves, *T. decolorata* was found from the entrance zone all to the aphotic zone. Individuals were found under rocks on humid or dry unconsolidated substrate, among roots, or rarely over bat guano (mixed guano of frugivorous and insectivorous bats). This species has also been recorded in anthropically disturbed areas in the nearby municipality of Parauapebas in Pará (Paulo Ricardo S. Coelho, pers. comm. 23 March 2026, species identity confirmed via photographs).

## Discussion

The numerous caves in Brazil have only recently begun to be explored more thoroughly for their gastropod fauna (Salvador et al. 2022) and are considered a promising source for new species discovery (Salvador 2019; Machado et al. 2023; Salvador et al. 2024). The present study is an initial step in studying the gastropod cave fauna in the northern region of Brazil.

Terrestrial gastropods were found in 15 out of the 31 caves explored in the present study (Fig. 1). A total of seven species were found, four Achatinidae and three Scolodontidae (although *Systrophia siolii* was found only in epigean areas, not inside the caves per se). Members of these two families seem to be the most common inhabitants of caves in Brazil, a trend observed by previous studies (e.g., Salvador et al. 2022, 2023), which is becoming somewhat of a rule as more caves are surveyed.

Still, the absence of other families in the in the Carajás caves is noteworthy, as they are often present (even if in small numbers) in other caves around the country, particularly the Helicinidae, Streptaxidae and Orthalicoidea. The latter represents the most diverse group in Brazil, and a few species can often be found in caves (e.g., Salvador et al. 2021, 2022, 2023).

This pattern may be related to geomorphology, as the caves in Carajás are within iron-rich and silica formations (Piló et al. 2015), while the remainder of the studies in Brazil have dealt with calcareous caves. Limestone environments are known to be favoured by land snails, often linked with higher diversity of species and/or higher abundance of individuals, given the importance of calcium availability for shell building (e.g., Hotopp 2002; Schilthuizen et al. 2003). However, considering that the studies of iron-rich caves in Brazil are still starting, it is too early to draw any firm conclusions.

Among all species found in this study, *Leptinaria unilamellata* and *Tamayoa decolorata* were the most frequently encountered, both in terms of number of specimens and number of occupied caves. They are common species in surface environments and are widely distributed, so that could be expected. Both new species described herein, as well as the potentially new *Dysopeas* sp., were recorded only in caves. As explained above for *Scolodonta carajaensis* sp. nov., an epigean population is suspected, so we consider it a troglophile (Trajano and Carvalho 2017). *Rectobelus arsayae* sp. nov. and *Dysopeas* sp. are known from shells only, which could have been transported into the caves, though we consider that unlikely due to the delicate structure of the shell; thus, for now, we also consider them as troglophiles.

This study also includes meaningful new records for some species and for Brazil. Firstly, it contains the first record of modern specimens (i.e., from a live population) of *Rectobelus* in Brazil; the previous record (Simone and D’ávila 2019) was based exclusively on archaeological material, so it remained unclear whether members of this genus could still be found in the country. Secondly, it is the first record of *Tamayoa decolorata* in Brazil, which, despite never having been detected so far, seems to be widespread and somewhat resistant to anthropogenically disturbed environments, as seen by its occurrence in an urban area in Carajás. Finally, there is a substantial range extension (>500 km) in the known geographic distribution of both *Leptinaria parana* and *Systrophia siolii* in Pará. Both these species remain endemic to Pará state, but it is worthwhile noting that this Brazilian state, with an area of ca 1,250,000 km^2^, is larger than France and the Iberian Peninsula combined.

During the present study, individuals of *Dysopeas* sp., *L. unilamellata* (Achatinidae) and *Tamayoa decolorata* (Scolodontidae) were found alive on bat guano (mixed frugivorous and insectivorous bats) in different caves. Guano is a known source of food within subterranean environments, and many cave invertebrates are dependent on it, either feeding directly off it or feeding on the many fungi that grow on it (Moulds 2004; Vanderwolf et al. 2013; Cunha et al. 2020). Still, very little has been reported on whether gastropods could use guano (and/or its fungal community) as a food source. Previous reports of snails occurring in guano habitats include one Australian Charopidae (Moulds 2004); one Japanese Euconulidae (Kawamura and Hayase 2025); and two exotic species introduced to the Antilles, including the ever-present *Subulina octona* (Bruguière, 1789), reported as being a “guanophage” (Peck 1971, 1974a, 1974b, 1975, 1981). While it is no surprise that the widespread anthropochoric *Subulina octona* can tap into a myriad of food sources, the evidence linking the other species to guano remains anecdotal. In Brazil, there is an arthropod-focused study on bat guano communities (Gnaspini and Trajano 2000) that lists a single land snail: an undetermined “Subulinidae” (now part of Achatinidae), which could well be *Subulina octona* (the species occurs in some Brazilian caves; e.g., Salvador et al. 2023). In the present study, we could not observe whether the live snails were actively eating the guano when collected. A comparative study of nitrogen isotope signatures of body tissues of the snails and samples of different types of guano would help clarify this matter.

Finally, the finding of two new species in the study area, which has mining activities despite the reserves, reflects recent discussions on the importance of targeting type localities in conservation, particularly in the face of the many environmental disasters that affected Brazil in the past decades (Azevedo-Santos and Ottoni 2025). The present case, in particular, has the added benefit of being an unusual combination of animal taxa (snails) and setting (ferruginous caves), which could improve the strength of arguments in favour of conservation management. Though Carajás already has many iconic animals, such as the Carajás woodcreeper (*Xiphocolaptes carajaensis* Silva, Novaes & Oren, 2002), *Scolodonta carajaensis* sp. nov., with its remarkable orange-red body, could be made into at least a minor flagship species for the area.

## Supplementary Material

XML Treatment for Rectobelus

XML Treatment for Rectobelus arsayae

XML Treatment for Dysopeas

XML Treatment for Leptinaria parana

XML Treatment for Leptinaria unilamellata

XML Treatment for Scolodonta carajaensis

XML Treatment for Systrophia siolii

XML Treatment for Tamayoa decolorata

## Additional information

### Artificial Intelligence (AI) use

The authors accept full responsibility for the content of the manuscript, including the disclosure of any use of AI.

No AI tools were used in the preparation of this manuscript.

### Author ORCIDs

R.B. Salvador https://orcid.org/0000-0002-4238-2276

L. Ferreira-Santos https://orcid.org/0009-0000-3805-0319

D.C. Cavallari https://orcid.org/0000-0003-3104-6434

J.E. Gallão https://orcid.org/0000-0002-5268-7946

M.E. Bichuette https://orcid.org/0000-0002-9515-4832

### Data availability

The authors confirm that the data supporting the findings of this study are available within the article. Genetic sequences are available from GenBank.
